# Hydrogel-By-Design: Smart Delivery System for Cancer Immunotherapy

**DOI:** 10.3389/fbioe.2021.723490

**Published:** 2021-07-21

**Authors:** Rongwei Cui, Qiang Wu, Jing Wang, Xiaoming Zheng, Rongying Ou, Yunsheng Xu, Shuxin Qu, Danyang Li

**Affiliations:** ^1^ Research Center, The Seventh Affiliated Hospital, Sun Yat-sen University, Shenzhen, China; ^2^ Key Lab of Advanced Technologies of Materials, Ministry of Education, School of Material Science and Engineering, Southwest Jiaotong University, Chengdu, China; ^3^ School of Life Sciences, Northwestern Polytechnical University, Xi’an, China; ^4^ Department of Gynaecology and Obstetrics, The First Affiliated Hospital of Wenzhou Medical University, Wenzhou, China; ^5^ Department of Dermatovenereology, The Seventh Affiliated Hospital, Sun Yat-sen University, Shenzhen, China

**Keywords:** hydrogels, smart delivery, cancer immunotherapy, immunomodualtors, immune cells, environmental regulatory substance

## Abstract

Immunotherapy has emerged as a promising strategy for cancer treatment, in which durable immune responses were generated in patients with malignant tumors. In the past decade, biomaterials have played vital roles as smart drug delivery systems for cancer immunotherapy to achieve both enhanced therapeutic benefits and reduced side effects. Hydrogels as one of the most biocompatible and versatile biomaterials have been widely applied in localized drug delivery systems due to their unique properties, such as loadable, implantable, injectable, degradable and stimulus responsible. Herein, we have briefly summarized the recent advances on hydrogel-by-design delivery systems including the design of hydrogels and their applications for delivering of immunomodulatory molecules (e.g., cytokine, adjuvant, checkpoint inhibitor, antigen), immune cells and environmental regulatory substances in cancer immunotherapy. We have also discussed the challenges and future perspectives of hydrogels in the development of cancer immunotherapy for precision medicine at the end.

## Introduction

In the past decade, cancer immunotherapy has shifted the paradigm for the treatment of cancers that it activates patients’ own immune systems to attack and kill cancers. Till now, cancer immunotherapy includes five main classes, immune checkpoint blockade (ICB) therapy, lymphocyte promoting cytokine therapy, chimeric antigen receptor T-cell (CAR-T) therapy, agonistic antibodies and cancer vaccines (Chao et al., 2019; Tang et al., 2021; Yang et al., 2020). The success of those therapies relies on the administration of corresponding biomacromolecules such as checkpoint inhibitors, cytokines, antibodies or T cells and vaccines (Li et al., 2020; Tang et al., 2021). However, high dose or multiple injections involved in conventional drug administration methods may introduce many problems such as safety issues and efficacy (Chao et al., 2019). For instance, the toxicity caused by the delivery agents and the complex tumor microenvironments are still challenges for the development of suitable delivery systems for cancer immunotherapy (Vigata et al., 2020). Therefore, novel delivery approaches could increase the accumulation of immune therapeutics in targeted sites, enabling more effective treatment and reduced side effects (Qiu et al., 2020). Biomaterials such as lipids (Yang et al., 2020), polymers (Munoz et al., 2021), polypeptides and hydrogels (Hoffman, 2012; Munoz et al., 2021) have played vital roles as smart delivery systems for cancer immunotherapy. The unique physicochemical properties of hydrogel have attracted broad attentions of researchers (Hoffman, 2012; Pal et al., 2013).

Firstly, hydrogels are formed by hydrophilic polymer chains absorbing large amounts of water (Ahmed, 2015; Oliva et al., 2017). The water content could reach 90%, which provides a suitable physiological environment for the cargoes (Akhtar et al., 2016; Xie et al., 2021). Hydrogels with excellent biocompatibility highly mimic the properties of nature tissues because of their high water content (Salah et al., 2020). Furthermore, the softness and rubbery properties of hydrogels minimize inflammatory reaction of surrounding cells. Especially for CAR-T therapy, cell viability could be guaranteed which is the basis for its immune function (Palucka and Banchereau, 2012; Seliktar, 2012; Monette et al., 2016; Zeng et al., 2021). Secondly, the availability of functional groups and the adjustable of the formed network structure of hydrogels allow carrying cargoes with various types and via different magnitude of interaction forces (Qiu et al., 2020). For example, Leach et al. developed an injectable multi-domain peptide-based hydrogel that can carry cyclic dinucleotides and STING (stimulator of interferon genes) agonists through electrostatic interactions (Leach et al., 2018). Additionally, the injectable properties of hydrogels through quick sol-gel phase transition or *in situ* chemical polymerization render them implantable in the body with minimal surgical wounds and the *in situ* entrapment of therapeutic molecules or cells by simple syringe injection at target sites (Overstreet et al., 2012; Leach et al., 2019; Vigata et al., 2020). Yu *et al.* summarized the status and development trend of injectable hydrogel-based cancer immunotherapy and claimed that injectable hydrogels have unprecedented potential to realize the inhibition of tumor growth at the systemic level and prevent the occurrence of side effects through local administration (Chao et al., 2019). Moreover, hydrogels respond immediately to various external stimuli and undergo morphological changes, which realize sustained and controlled release of therapeutics for cancer immunotherapy (Lin and Metters, 2006; Ma et al., 2021). Novel pH-responsive hydrogels with high swelling property at acidic pH were shown to release drug at a relative fast rate by adjusting the ratio of chitosan and alginate polymers with poly (vinyl alcohol) (Andrade et al., 2021). In addition, the advantages of ease of synthesis, and low raw material prices also make hydrogels a very promising delivery agent for multiple immunological therapeutics (Ahmed, 2015).

This review concentrates on the state-of-the-art of hydrogels applied in cancer immunotherapy as efficient, biocompatible, controllable local drug delivery platforms. The following sections discussed the design principles of hydrogels for delivery therapeutics for cancer immunotherapy, which include the choice of polymer feedstock, the strength of the interaction force between the polymer network, the polymer modification etc. In addition, specific applications of hydrogels in cancer immunotherapy that classified by the carrying cargos which includes cytokines, adjuvant, checkpoint inhibitors, antigen, cells and environmental regulatory substance were discussed and summarized. At last, the development trend, main challenges and clinical application potential of hydrogel-based delivery systems for cancer immunotherapy were discussed.

## Characteristics of Hydrogels as Delivery Systems for Cancer Immunotherapy

Cancer immunotherapy mainly aims to achieve the following purposes, e.g., enhancing the functions of antigen presenting cells, promoting the production of protective T-cells and overcoming immunosuppression (Zou, 2006; Tang et al., 2021). The corresponding therapeutics commonly used in cancer immunotherapy, such as biologically antigenic proteins or peptides, nucleic acids, natural or synthetic adjuvants, and immune cells require the delivery systems to retain their activities and functions (Zhang et al., 2018; Chao et al., 2019).

**FIGURE 1:**
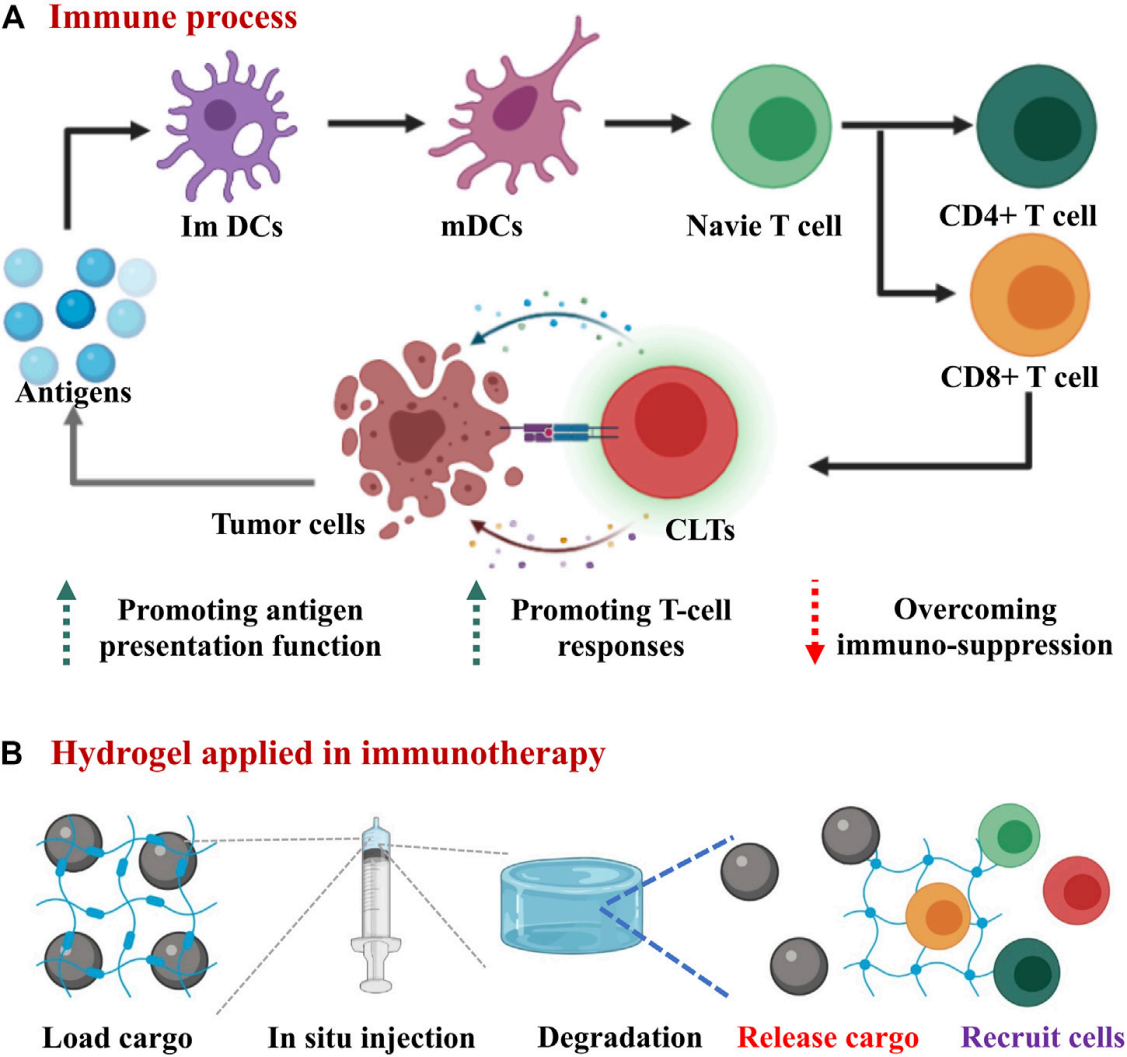
Overview scheme of hydrogels applied in cancer immunotherapy. **(A)** The schematic diagram of the immune response in the body: antigens are processed by immature dendritic cells (ImDCs), then mature dendritic cells (mDCs) presented antigens to T cells. CD8^+^ T cells further differentiate into cytotoxic T lymphocytes (CTLs) to directly kill tumor cells. **(B)** hydrogels applied in immunotherapy: the hydrogel carrying various cargos is injected into the tumor site and then undergoes degradation, thereby releasing drugs and recruting immune cells to tumor site.

**FIGURE 2:**
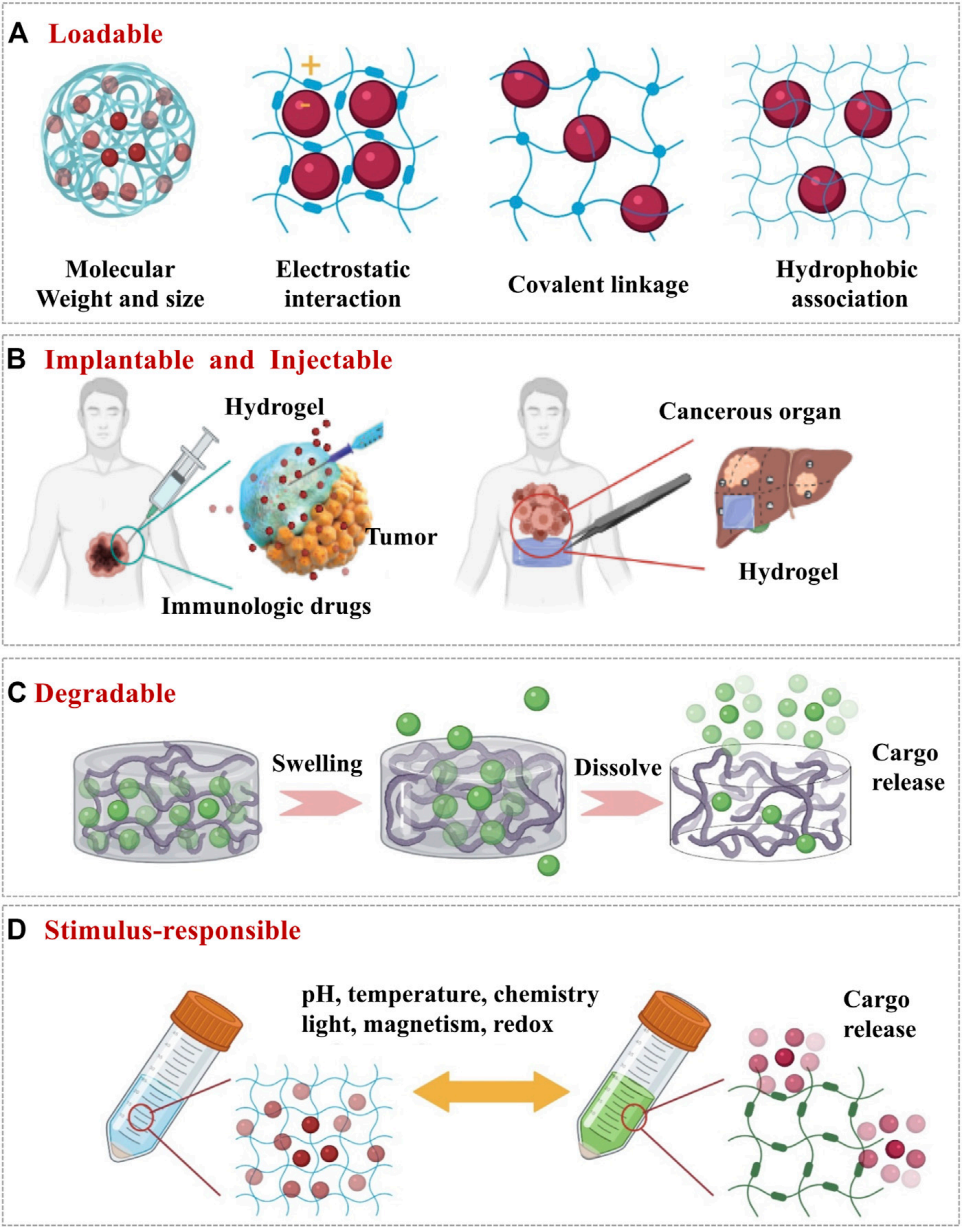
The design of hydrogel by altering its physicochemical properties as delivery systems for cancer immunotherapy. **(A)** Loadable: ensure that the immune cargo can stay in the hydrogel when it does not need to be released. **(B)** Implantable and injectable: ensure that the hydrogel can reach the tumor site in a minimally invasive, non-surgical way and stays at the tumor site for drug release. **(C)** Degradable: hydrogel degrades for releasing the encapsulated drugs. **(D)** Stimulus-responsible: to achieve controlled release of the drugs from hydrogel upon external stimuli.

Hydrogel is a particularly appealing type of drug delivery system because of its unique characters, providing the delivered immunological therapeutics with a physiological environment that highly mimics the natural conditions (Figure 1; Hori et al., 2008; Hickey et al., 2019). These desirable properties include loadable (Figure 2A), implantable/injectable (Figure 2B), degradable (Figure 2C) and stimulus-responsible (Figure 2D). Hydrogels as drug delivery systems can be divided into two categories according to their component sources: synthetic and natural (Chao et al., 2019). Commonly used synthetic hydrogels are hydroxyethyl methacrylate (pHEMA), poly(vinyl alcohol) (PVA), and poly(ethylene glycol) (PEG). These components often have stimulus response characteristics such as temperature sensitivity, so that the hydrogel can be polymerized or degraded *in situ* (Rizzo and Kehr, 2021). Natural hydrogels include polysaccharides (such as hyaluronic acid and alginate), proteins (such as gelatine and collagen), and DNA. This type of hydrogel has extremely high biocompatibility. The large number of hydroxyl, carboxyl and amino groups in the composition also makes it capable of loading a variety of drugs (Choudhury et al., 2018; Hauptstein et al., 2020). In this part, we focused on the key characters of hydrogels influencing the delivery of therapeutics for cancer immunotherapy.

### Loadable

First of all, hydrogel has the capability to easily encapsulate hydrophilic drugs due to its high water content. The water content of the hydrogel is related to the type of polymers. Natural polymers such as hyaluronic acid-based hydrogel can achieve 99% water content (Tu et al., 2019; Dorishetty et al., 2020). Secondly, the crosslinked network of hydrogel could prevent the drugs from leaking out and impede penetration of various proteins that may degrade the loaded biological drugs (Pal et al., 2013). This feature means the hydrogel can be loaded with highly labile macromolecular drugs (for example, recombinant proteins and monoclonal antibodies). According to David J. Mooney, the space between polymer molecules in the network (referred to as the mesh size) is tunable from around 5 to around 100 nm (Li and Mooney, 2016). Importantly, the network can retain the drugs in the “mesh” before the state of the hydrogel changes. The mesh size of hydrogels can be adjusted by the molecular weight and concentration of the polymer (Figure 2A). In general, the higher the molecular weight and the concentration, the smaller the mesh size can be achieved to encapsulate the smaller size of drugs. Finally, there are various interactions (Figure 2A) between the drugs and the polymer chains which could be designed *via* diverse strategies such as covalent conjugation, electrostatic interactions and hydrophobic associations (Li and Mooney, 2016). The interactions between hydrogels and drugs are important to maintain the functions and activities of the loaded cargos when designing hydrogels. For instance, Dror *et al.* claimed the bioactivities of hydrogels could be achieved through immobilizing growth factors and/or small molecules such as RGD peptide (Arginine-Glycine-Aspartic acid) to the backbone polymer. With facilitainge the interactions with cellular and extracellular biomolecules, the hydrogel can also carry immune cells for therapeutic purposes(Chao et al., 2019; Li et al., 2020).

### Implantable

Immunotherapeutics on their own typically require repeated high-dose injections that often result in off target effects with limited efficacy. Implantable scaffolds formed by hydrogel possess powerful synergies with anti-cancer immunotherapies because they allow for discrete localization and controlled release of immunotherapeutic agents, which have been shown various applications to improve outcomes in the treatment of cancers *via* immunotherapy (Buwalda et al., 2014; Rizzo and Kehr, 2021). Among various strategies, implantable hydrogel can form robust tablet-like scaffolds that are surgically implanted into a tumor resection site, releasing immunoregulatory “cargos” and recruiting immune cells into the porous matrix of the scaffold for further biological programming (Li et al., 2020). In order to prepare hydrogels into implantable scaffold with different mechanical, degradation, and slow-release properties, the cross-linking process needs to be adjusted. Because of the covalent bonds presented between the polymer chains, chemical cross-linking is one of the highly resourceful methods for the formation of hydrogels with excellent mechanical strength (Tie et al., 2020). The hydrogel formed in this method usually degrades slower, so that the release rate of the carried immune drug is relatively slow. Simultaneously, literatures show that hydrogel with a large elastic modulus is beneficial for adhesion and spread of immune cells on its surface, while not being conducive for migration of immune cells into its three-dimensional interior of the hydrogel (Seliktar, 2012; Ahearne, 2014; Simona et al., 2015). Since physical cross-linking is relatively mild and avoids the use of cross-linking agents, it is much more friendly to loaded immunotherapy-related biologically active components such as cytokines and immune cells. The interaction forces formed between physical cross-links include hydrogen bonds, hydrophobic interactions and ionic interactions. These weak interaction forces are extremely easy to be destroyed, which makes the drug delivery system easy to degrade and results in relatively rapid drug release (Buwalda et al., 2014). The most advanced application of implantable hydrogels in immunotherapy is enhance adoptive T cell (ATC) immunotherapy because it is able to support T cell viability and localize the desired immune response (Oh et al., 2016). Stephan *et al.* synthesized a calcium cross-linked alginate scaffold containing synthetic collagen mimic peptides, which can promote the adhesion and migration of T cells and also increase the expansion and dispersion of T cells to the tumor resection bed (Hori et al., 2008; Rastogi and Kandasubramanian, 2019).

### Injectable

Injectable hydrogels formed *in situ* by chemical polymerization or sol–gel phase transition have been widely concerned in drug delivery and cancer immunotherapy (Chao et al., 2019). Injectable means the materials are flowable before injection but rapidly turn into gels once injected (Figure 2B). Such materials have been widely used because of a few distinct advantages (Rizzo and Kehr, 2021). Firstly, they can be positioned and delivered following the needle, which is a simpler and less traumatic process that avoids unnecessary tissue damage and complications related to inflammation. Secondly, with their viscoelastic properties, injectable materials can move and flow, conforming to any available space before forming into a persisting implant. Koshy *et al.* reported an injectable gelatin cryogels derived from natural collagen, which were shown to be easily administered without a surgical procedure, promoting infiltration and proliferation of immune cells with controlled release of granulocyte-macrophage colony-stimulating factor (GM-CSF) (Duong et al., 2020). Thirdly, it is easy to encapsulate cells or drugs.

At present, rapid construction of injectable and biocompatible hydrogels with proper mechanical properties remains a challenge. Many methods including thermal gelation, ionic interaction, physical self-assembly and photopolymerization have been employed for preparation of injectable hydrogels *in situ* (Lei and Tang, 2019; Duong et al., 2020). The “click” reaction, with easy preparation and rapid reaction rate, has emerged as one of the most versatile and innovative technologies (Lei and Tang, 2019). Due to the fast reaction rate and mild reaction conditions, click chemistry is often used to fabricate bio-orthogonal hydrogels, which are highly suitable as carriers for delivering immunotherapy related drugs. In a study by Xu *et al.*, two types of biodegradable and injectable hydrogels using thiol–Michael addition reaction were found to be capable of carrying cells and allowing them function when reaching the designated site (Liu et al., 2020). In addition, Brudno *et al.* confirmed that utilizing biorthogonal click chemistry to link the functionalized hydrogel with modified anti-cancer pro-drug agents *in situ* (Ma et al., 2021). In clinical immunotherapy, injectable and implantable can exist simultaneously once the hydrogel is delivered to the tumor site through the needle, the hydrogel can spontaneously form a high-strength scaffold. Kim *et al.* have developed an injectable cancer vaccine scaffold based on mesoporous silica rods. The system is an ingenious compromise between implantable stents and injectable biomaterials. When injected subcutaneously, it self-assembles into a unique pocket-like reservoir *in situ*, allowing extended load factor release (Chao et al., 2019).

### Degradable

When used as drug delivery system in cancer immunotherapy, hydrogels mainly release drugs at tumor sites in two ways: hydrogel degradation and hydrogel swelling (Oliva et al., 2017). As the network degrades, the mesh size increases, allowing drugs to diffuse out of the hydrogel (Figure 2C). Hydrogels undergo local or bulk dissolution *via* a number of mechanisms (such as hydrolysis, proteolysis, disentanglement, or environmental triggers), whilst degradation can occur in the polymer backbone or at the crosslinks, and is typically mediated by hydrolysis or enzyme activity (Tan and Marra, 2010; Vojtova et al., 2019). The hydrogel can be passively degraded or can be actively degraded in response to a certain stimulus, achieving the controlled release of immune “cargos”, which will be discussed in more detail in the next section. Furthermore, the degradation process should also not be too short nor too long because the short degradation of hydrogels will cause the explosive release of the drugs and long degradation which will inactivate the immune drugs or cause inflammation in the body. On the other hand, the biodegradation of hydrogels is essential for biomedical applications where controlled *in vivo* absorption and/or local lysis is required to facilitate cell movement and morphogenesis. For biodegradability, the most common method is to introduce labile bonds in hydrogels either in the cross-links, or the network backbones. Unstable linkages may be cleaved in physiological conditions, either chemically or enzymatically, typically by hydrolysis. Hu *et al.* reported that a hyaluronic acid hydrogel carries PD-L1antibody (aPD-L1) which is grafted onto the platelet. When the hydrogel is injected into the tumor site, inflammation will initiate platelet activation and release aPD-L1, which achieves a controlled release (Hu et al., 2021).

### Stimulus-Responsible

Those hydrogels with stimulus-responsive properties can be used as a drug delivery system in cancer immunotherapy, in which drug release is triggered upon external stimulis (Bajpai et al., 2008; Andrade et al., 2021) such as pH, temperature, and biomolecule concentration (Figure 2D). The cross-linking process of the hydrogel can respond to external stimuli. One of the most representative example is the novel thermogelling poly(lactic-co-glycolic acid)–poly(ethylene glycol)–poly(lactic-co-glycolic acid) (PLGA−PEG−PLGA) triblock copolymer, which exhibits a sol−gel transition with increasing temperature (Li et al., 2021). Jiang *et al.* incorporated multifunctional dendritic nanoparticles into a injectable thermosensitive hydrogel matrix (PLGA-PEG-PLGA triblock copolymers) to construct a localized drug delivery system for combining chemotherapy and immunotherapy (Vojtova et al., 2019). It is noteworthy that the dendritic nanoparticles-in-hydrogel delivery system gels at physiological temperature, thus allowing for sustained release of drugs. Ultimately, this system showed remarkable efficacy in treating triple negative breast cancer in mice model with 86% tumor growth inhibition. Additionally, the degradation process of hydrogel can respond to external stimuli. In response to pathological cues, hydrogel undergoes degradation upon external stimuli, followed by the release of the drugs which was precisely controlled by the pathological need. Gajanayake *et al.* developed a hydrogel that can release the immunosuppressant tacrolimus responsively to deal with proteolytic enzymes overexpressed in inflammation. Within 14 days, drugs in the hydrogel can be completely released, while the drug release rate in phosphate buffered saline for 28 days is less than 10%. Immunosuppressive drug tacrolimus was encapsulated in hydrogelS responsive to proteolytic enzymes which are overexpressed in inflammation. Within 14 days, complete drug release was achieved without burst release, whilst the drug release in phosphate-buffered saline was below 10% over 28 days (Oliva et al., 2017). The swelling behaviour of hydrogels can be affected by various external physicochemical conditions, which have been widely exploited in controlled drug delivery. pH responsive swelling is particularly important for cancer delivery systems because it allows targeted drug release in solid tumors where tumour microenvironments are typically more acidic than normal tissues (Andrade et al., 2021). Other stimuli responsive swelling mechanisms have also been exploited for controlled drug delivery. For example, a temperature responsive nanogel was fibricated to deliver the chemotherapy drug cisplatin to breast cancer that the temperature was believed to be slightly higher than the normal ones (Li et al., 2021) In some other cases, the drug carried by the hydrogel responds to stimuli. Natalie *et al.* incorporated gold nanoparticles decorated with 5-fluorouracil (5-FU)-hairpin DNA into dendrimer-dextran hydrogels to silence multidrug resistance protein-1 (MRP1). When the hairpin is opened by hybridizing with a complementary target (MRP1 mRNA), this interaction becomes weaker, allowing simultaneous release of the drug and knockout of the gene encoding the protein responsible for drug resistance (Ding et al., 2019). These smart properties of hydrogels mentioned above make hydrogel attractive and praisable as drug delivery systems for cancer immunotherapy.

## Applications of Hydrogels as Delivery Systems for Cancer Immunotherapy

### Delivery of Immunomodulatory Molecules

**FIGURE 3:**
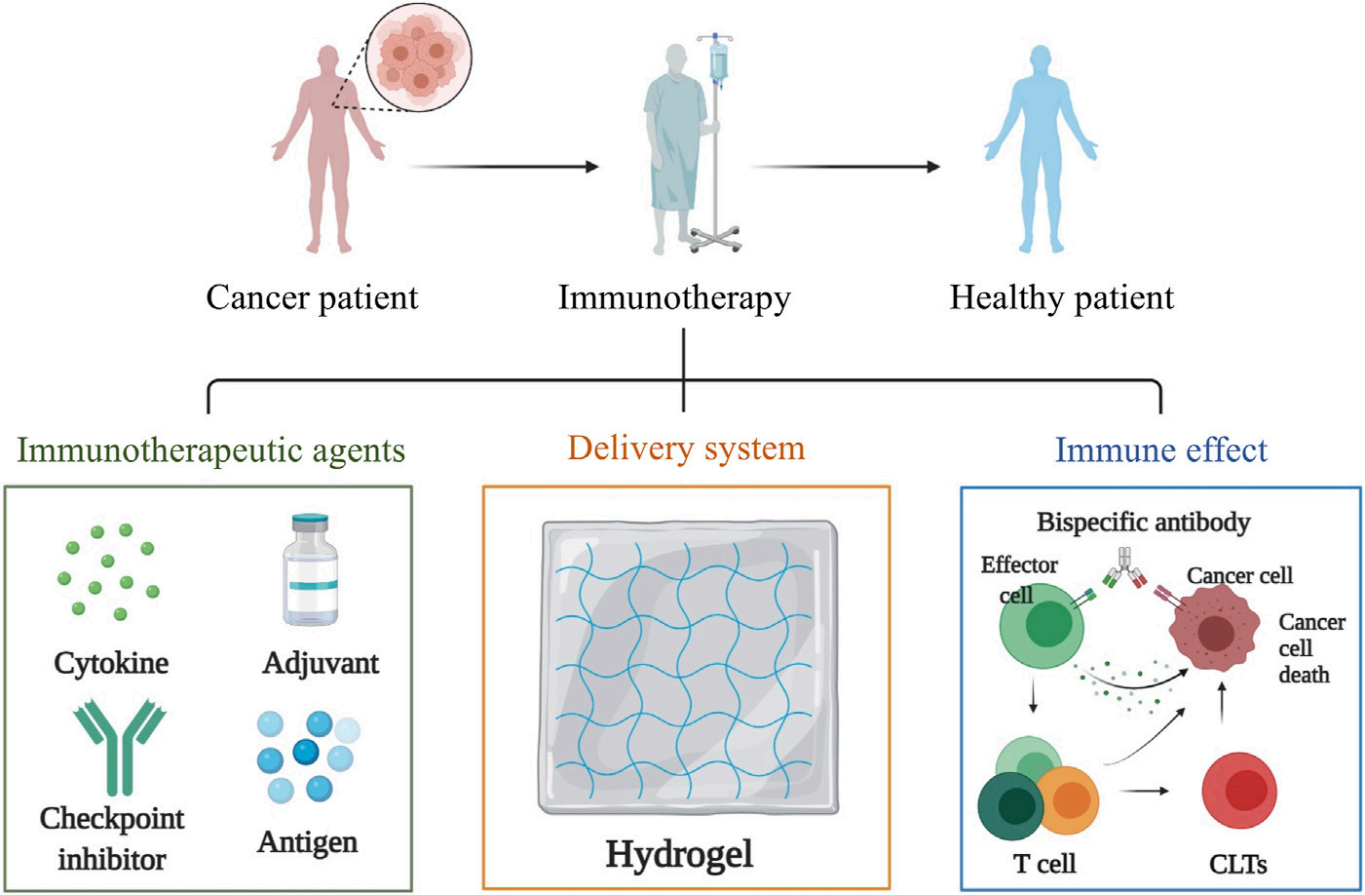
Hydrogels applied in the delivery of immunomodulatory molecules which include cytokine, adjuvant, checkpoint inhibitor and antigen. These immunomodulatory molecules can be loaded to the biocompatible hydrogel in a simple and gentle way to ensure the biological activity of these immunomodulatory factors, enabling them to exert their immune effects.

A wide variety of immunotherapeutic agents, from small molecule drugs to macromolecular drugs and even cells, can be incorporated into the hydrogel for enhanced cancer immunotherapy. In this section, we discussed the application of hydrogel as delivery system for immunomodulatory molecules such as cytokine, adjuvant, checkpoint inhibitor and antigen (Figure 3).

### Cytokine

In cancer immunotherapy, cytokines can be used to either activate or suppress the immune response against tumors (Chao et al., 2019). For example, Interleukin-12 (IL-12), a heterodimeric cytokine composed of two polypeptides with disulfide linkage, enhances the immune response of Th1, the maturation of cytotoxic T lymphocytes, the stimulation of natural killer cells, and the production of interferon-γ (IFN-γ). To overcome the main impediment of cytokines’ short half-life *in vivo*, Shimizu *et al.* encapsulated IL-12 in a cholesterol-bearing pullulan (CHP) nanogel. After the mice were subcutaneously injected with murine IL-12-loaded CHP nanogel (CHP/rmIL-12), the concentration of serum rmIL-12 stays relatively high for 12–24 h (Shimizu et al., 2008). GM-CSF, also known as colony stimulating factor 2 (CSF2), is a monomeric glycoprotein that secretes by macrophages, T cells, mast cells, natural killer cells, endothelial cells, and fibroblasts. Doo Sung Lee *et al.* have designed an injectable smart hydrogels composed of levodopa and poly(ε-caprolactone-co-lactide)ester-functionalized hyaluronic acid (HA-PCLA) that loaded with OVA expressing plasmid (pOVA) and GM-CSF to boost the maturation of dendritic cells (DCs). By the controlled release of pOVA and GM-CSF through a single subcutaneous injection, the hydrogel system effectively eliminated the B16/OVA melanoma tumors (Leach et al., 2018).

### Adjuvant

Chemotherapy and radiotherapy can induce immunogenic cell death (ICD), such antitumor immune response would be further amplified if there are concurrent immune adjuvants inside the tumor. Adjuvants are nonspecific immunostimulants, which enhance the body’s immune response when combined with antigens. For example, Hou *et al.* used genetically engineered polypeptide hydrogel PC10ARGD as a sustained-release system that delivered Bestatin which is an immune adjuvant for mammary carcinoma treatment. The hydrogel serves as a drug delivery depot for sustained drug release of Bestatin, which further inhibits tumour metastases and microvessel formation but amplified T lymphocytes function (Hou et al., 2020). Liu *et al.* developed a smart hydrogel based on alginate, which hybridized with immunoadjuvant CpG oligonucleotide and conjugated with an adenosine triphosphate (ATP)-specific aptamer. Once intratumoral injection, this hydrogel gelled *in situ* and released the CpG oligonucleotide, achieving remarkable synergistic tumor elimination as well as generating long-lasting immune memory to prevent recurrences (Wu et al., 2021).

### Checkpoint Inhibitor

Immunological checkpoints are a class of immunosuppressive molecules that can regulate the intensity and duration of immune responses, thereby avoiding damage of normal tissues. During the occurrence and development of tumors, immune checkpoints become one of the main regulators for immune tolerance (Yu et al., 2018). Immune checkpoint blockade therapy is an approach that regulates the activity of T cells through a series of channels such as cosuppression or costimulation signals to kill tumor cells. The most commonly used therapeutic drugs are programmed death receptors 1/programmed death receptor-ligand 1 (PD-1/PD-L1) and cytotoxic T lymphocyte-associated antigen-4 (CTLA-4) inhibitors. Activated T cells express PD-1 to recognize and remove abnormal or cancerous cells. However, tumor cells inactivate the T cells that recognize tumor antigens by expressing PD-1 ligands that bind to PD-1, thereby evading immune system attacks. Therefore, tumor cell death can be induced by blocking inhibitors of PD-1 or its ligand. CTLA-4 is another immune checkpoint that reduces T-cell activation and promotes tumor progression by binding to its ligands. Inhibitors against CTLA-4 and its ligands block their interactions to increase T-cell activity and thereby clear tumors (Kazemi et al., 2021; Tang et al., 2021). Immune checkpoint inhibitors can cause side effects to many normal organs. Therefore, a large number of studies have used hydrogel as delivery system to locally release immune checkpoint inhibitors at the tumor site to solve the above problems. Chen *et al.* developed an anti-inflammatory nanofiber hydrogel self-assembled by steroid drugs for local delivery of (αPD-L1). Effective therapeutic effects were observed with inhibition both local tumors and abscopal tumors once injected with αPDL1-loaded hydrogel.

### Antigen

Low immunogenicity and tumor heterogeneity greatly limit the therapeutic effect of tumor vaccines. In view of this, Zhu *et al.* prepared a new type of injectable adhesive hydrogel based on thermosensitive nanogels containing catechol groups and loaded with MnO_2_ nanoparticles. After intratumoral injection, the concentrated nanogel dispersion transformed into an adhesive hydrogel *in situ*. The photothermal effect of the loaded MnO_2_ nanoparticles induces immunogenic cell death and releases a large amount of autologous tumor-derived protein antigens under near-infrared radiation. These antigens captured by the hydrogel are ideal immunostimulatory substances to avoid the consequences of tumor heterogeneity. They also recruit more dendritic cells to stimulate a strong and durable anti-tumor immune response mediated by CD8^+^ T cells (Song et al., 2018). Wang *et al.* used polypeptide hydrogel loaded with tumor cell lysates (TCL) as antigens, Toll-like receptors 3 (TLR3) agonist, polyinosinic:polycytidylic acid [poly(I:C)] to construct the vaccines, which robustly recruit, activate and mature DCs *in vitro* and *in vivo* by sustained release of TCL and poly (I:C). The antigen persistence at the injection site and antigen drainage to lymph nodes were significantly improved by hydrogel (Song et al., 2018).

### Delivery of Immune Cells

**FIGURE 4:**
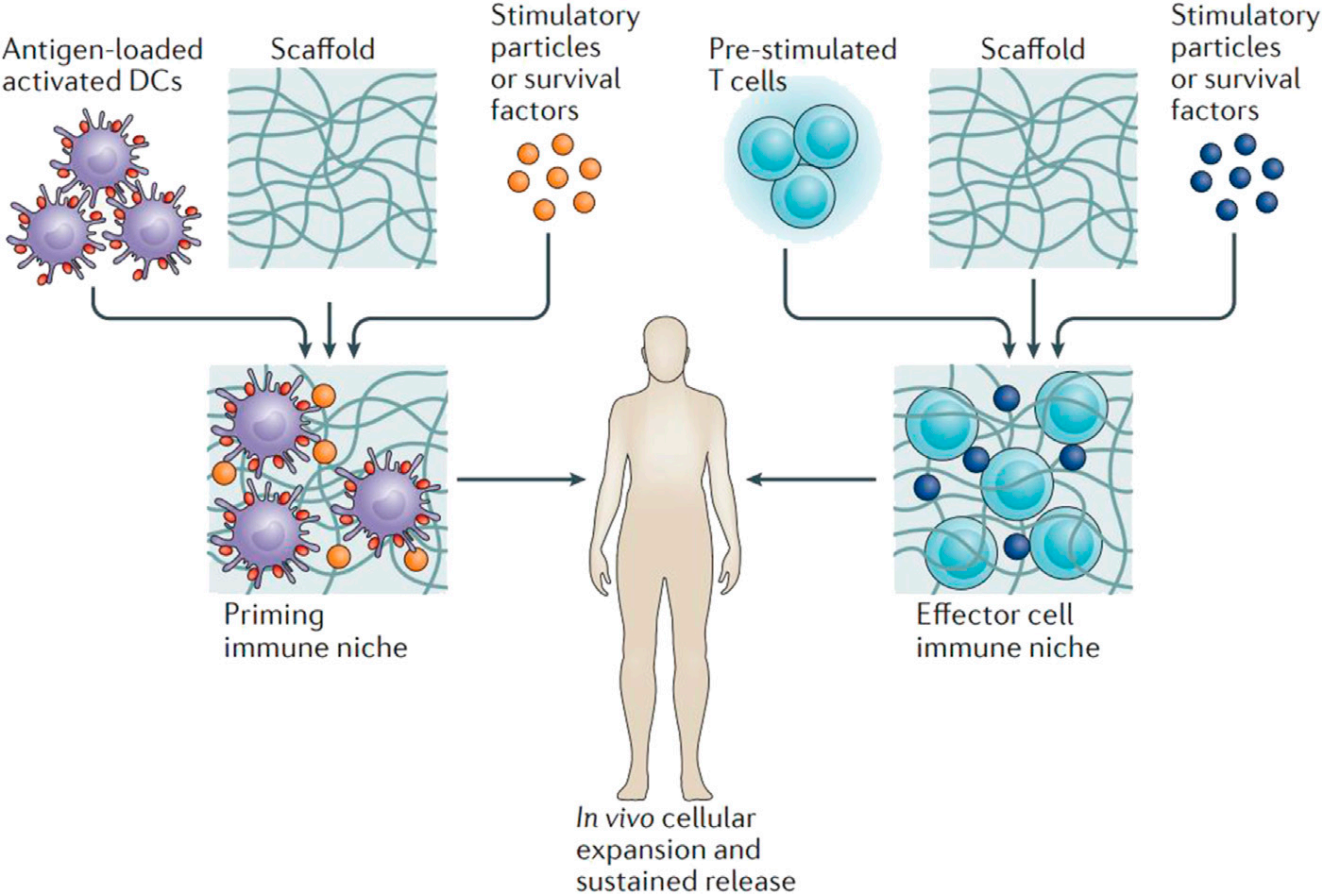
Hydrogels applied in the delivery of immune cells. Antigen-loaded activated dendritic cells or pre-stimulated tumour-specific T cells are loaded to hydrogel scaffolds *ex vivo*. Stimulatory agents were encapsulated to support cell survival, activation and expansion. After administration of the matrix close to the tumour site, potent immune cells proliferate within the scaffold and are released continuously into the tissue environment. Reproduced with permission Weiden et al. (2018). Copyright 2018 Nature.

Localized delivery using hydrogels in immunotherapy provides adjustable microenvironments for immune cells as it enables the recruitment, expansion, and activation of immune cells *in vitro* and *in vivo* (Figure 4; Weiden et al., 2018; Yang et al., 2018) It is reported that a thermosensitive chitosan hydrogel for the delivery of tumor-infiltrating lymphocytes and activated CD8^+^ T cells**.** To provide a suitable physical condition for the proliferation of immune cells, the gelation process and mechanical strength of the hydrogel could be precisely tuned by varying the concentration of sodium bicarbonate. The *in vitro* T-cell expansion experiment revealed that hydrogel platform with a larger pore size and higher stiffness promoted the viability and proliferation of the encapsulated T cells (Hickey et al., 2019). DCs can either be activated in hydrogels *in vitro* before implantation, or by immobilizing stimuli within the gels *in vivo* (Yang et al., 2018). Verbeke *et al.* designed an injectable alginate hydrogel system that allows DCs to be enriched locally *in vivo* without activation or maturation. Similarly, alginate hydrogels was designed to carry and releas antigen-loaded DCs when injected subcutaneously in mice (Hori et al., 2008). Besides DCs, macrophages also playws a major role as antigen-presenting cells in tumor vaccination. Muraoka *et al.* developed a cholesteryl pullulan-based hydrogel to deliver peptide-based antigens to macrophages (Chao et al., 2019). The antigens were successfully delivered to CD8^+^ cytotoxic T cells by this immunologically inert hydrogel.

Passive immunotherapy uses components of immune system to target specific cancers without necessarily involving an immune response. Since the first clinical trial 28 years ago, tumor-infiltrating lymphocytes (TILs) have been widely applied for the treatment of metastatic melanoma. Meanwhile remarkable research efforts have been focusd on adopative cell therapy (ACT), which heavily relies on the administration of allogeneic T cells (Monette et al., 2016). Furthermore, genetically modified tumor-specific T cells have been developed to overcome the limitation of TIL expansion and the augment ACT-mediated immunotherapy. Compared with systemic delivery, which is likely to result in T cells infiltrating to noncancerous tissues, localized administration using hydrogels into tumor sites improves delivery efficiency and sustained release of T cells. The feasibility of a thermosensitive, amine-reactive oligo(ethylene glycol) methacrylate-based hydrogel was investigated as a T cell carrier. Additionally, an injectable thermogel based on chitosan was developed with the purpose of encapsulating, expanding, and delivering cytotoxic T cells (Jiang et al., 2018).

### Delivery of Environmental Regulatory Substances

**FIGURE 5:**
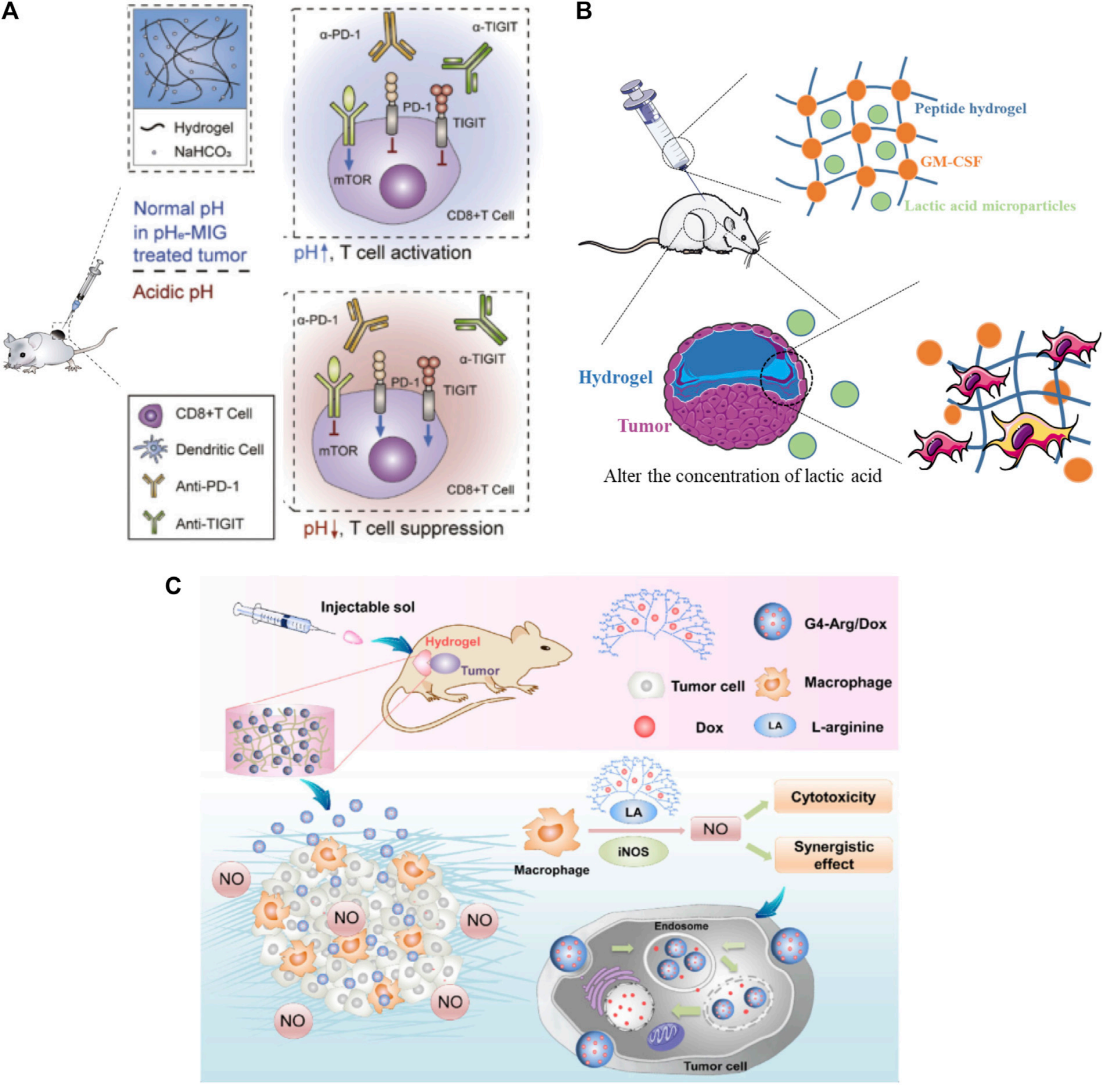
Hydrogels applied in the delivery of environmental regulatory substance. **(A)** Pluronic F-127 was used as a NaHCO_3_ releasing carrier to focally alleviate extracellular tumor acidity. Reproduced with permission Jin et al. (2019). Copyright 2019 Elsevier Ltd. **(B)** A lactic acid-producing hydrogel system was design to change the concentration of lactic acid at the tumor site to interrogate immune cell modulation in cancer-like environments. Reproduced with permission Allen et al. (2020). Copyright. 2020. The Royal Society of Chemistry. **(C)** A dendritic scaffolds were designed to enrich arginine molecules and provide the inducible nitric oxide synthase (iNOS) substrate, L-Arg, to M1 macrophages, which can produce the cytotoxic substance nitric oxide (NO) and subsequently induce tumor cell destruction through immunotherapy. Reproduced with permission Jiang et al. (2018). Copyright 2018 springer.

Practically, in addition to carrying the above-mentioned cytokines, immune adjuvants, checkpoint inhibitors, and immune cells that directly stimulate immunity, the hydrogel can also carry environmental regulation substances to regulate the tumor microenvironment. For example, acidic extracellular pH promotes immune evasion and tumor progression. Therefore, antagonizing tumor acidity can be a powerful approach in cancer immunotherapy. Hyung-seung *et al.* used Pluronic F-127 as a NaHCO_3_ releasing carrier to focally alleviate extracellular tumor acidity (Figure 5A). In a mouse tumor model, intratumoral treatment with pH modulating injectable gel (pHe-MIG) generates immune-favorable TME, as evidenced by the decrease of immune-suppressive cells and increase of tumor infiltrating CD8^+^ T cells (Jin et al., 2019). It has also been suggested that implantable hydrogel can be engineered to investigate specific aspects of the tumor microenvironment, either singularly or in combination. It is known that lactate, a metabolite byproduct of anaerobic glycolysis, is known to reprogram immune cells, resulting in increased tumor survival. Riley *et al.* (Figure 5B) designed and synthesized a peptide hydrogel loaded with GM-CSF and poly-(lactic-co-glycolic acid)/(lactic acid) microparticles which can generate the localized lactate concentrations (∼2–22 mM) and cellular makeup of the tumor microenvironment, following subcutaneous implantation in mice and causing significant immunological effects (Allen et al., 2020). Jiang *et al.* incorporated multifunctional dendritic nanoparticles into a injectable hydrogel matrix which is composed with thermosensitive triblock copolymers to construct a localized drug delivery system for combining gas-therapy and immunotherapy (Figure 5C). The dendritic scaffolds were used to deliver arginine-rich molecules (L-Arg) to M1 macrophages, which produce the cytotoxic substance nitric oxide (NO), and subsequently induce tumor cell destruction (Jiang et al., 2018).

## Conclusion and Perspectives

Immunotherapy is a type of approach that utilizes the body’s own immune system to combat cancer, demonstrating to be effective or completely alleviating some solid or hematological malignant tumors. Hydrogels exhibited excellent promise for cancer immunotherapy since they are loadable, implantable, injectable, biodegradable and stimuli-responsive (Bu et al., 2019). These properties guarantee hydrogels higher immunological drug loading efficiency and drug stability, more controllable drug release profiles, and easier to combine immunotherapy with other treatment methods (Yang et al., 2020). In this review, we focus on how the characteristics of hydrogels have been applied to deliver cytokines, adjuvants, checkpoint inhibitors, antigens, cells, and environmental regulatory substance in cancer immunotheraoy. These reported hydrogel systems are able to prolong the residence time of immunotherapy factors at tumor sites, as well as protect bioactive molecules from denaturation or degradation, thus improving the efficacy of immunotherapy.

In spite of the successful exploration of hydrogel drug delivery systems for cancer immunotherapy, several challenges still need to be considered for the future development and potential applications. Firstly, the controlled release of drug from hydrogels still remains as an issue since immunological drug are released by diffusion and hydrogel degradation, both of which are uncontrollable processes. Those hydrogels are responsive to stimulation can release immunological drugs, but the research in this area is only limited to *in vitro* experiments or rely heavily on the intrinsic tumor microenvironment. Once the hydrogel implanted into the body, it will be important to find out if same type of response properties exists *in vivo*. Secondly, although hydrogel materials have demonstrated good biocompatibility and biodegradability, higher biocompatibility is needed when used to deliver immunological drugs. In clinical application, the safety of the delivery agents is extremely crucial, particularly in cancer immunotherapy, those bioactive molecules or cells discussed above require an environment that is similar to physiological conditions more than other drugs. Thirdly, there are some issues that need to be considered in clinical aspects: 1) multiple imaging-guided injection techniques should be developed for deep-sited tumors administration; 2) The dose of immune drugs is also a vital parameter. For *in vitro* experiments, a drug dose is usually chosen based on the effectiveness of the drug. In clinical practice, however, a strict dose screening should be performed according to the drug release curve of the hydrogel. High concentration of anticancer agents can also cause severe acute injury to the healthy parenchyma due to the burst release. If the drug release from the hydrogel does not proceed at a uniform rate, it can also lead to increase the risk of drug resistance; 3) It will be feasible to construct the final system on the basis of approved clinical materials as it is also more likely to get clinical approval.

In summary, the delivery systems based on hydrogels have exhibited excellent promises for cancer immunotherapy as the unique characteristics of hydrogels ensure locally and sustainably delivering immunotherapeutic agents to target tumor sites with reduced drug dose and side effects. However, challenges still exist, which requires experts from multidisciplinary fields work more closely. We believe this smart delivery system will continue contributing in tomour threatment *via* immunotherapy as well as other aspects for improving human health.
